# Randomized, double-blind, placebo-controlled trial of aripiprazole oral solution in children and adolescents with Tourette’s disorder

**DOI:** 10.1186/s13034-024-00764-6

**Published:** 2024-07-18

**Authors:** Fan He, Jie Luo, Yi Huang, Yunpeng Hao, Ling Sun, Xiaoyan Ke, Bin Wu, Yucai Chen, Ying Han, Yuebing Zhang, Jing Liu, Hong Han, Mingji Xian, Motomichi Uki, Yi Zheng

**Affiliations:** 1 Beijing Anding Hospital, Capital Medical University, Beijing, China; 2 West China Hospital, Sichuan University, Chengdu, China; 3 The First Hospital of Jilin University, Changchun, China; 4 Tianjin Mental Health Centre, Tianjin Anding Hospital, Tianjin, China; 5 Nanjing Brain Hospital, Nanjing, China; 6 Xi’an Mental Health Center, Xi’an, China; 7 Shanghai Children’s Hospital, Shanghai, China; 8 Peking University First Hospital, Beijing, China; 9 Shandong Daizhuang Hospital, Jining, China; 10 Peking University Sixth Hospital, Beijing, China; 11 Shanxi Children’s Hospital, Taiyuan, China; 12 Otsuka Beijing Research Institute, Beijing, China; 13 Otsuka Pharmaceutical Co., Ltd., Tokyo, Japan

**Keywords:** Aripiprazole solution, Tourette’s disorder, Tics, Adolescents, Children

## Abstract

**Background:**

Aripiprazole is the most frequently recommended antipsychotic for the treatment of tics in children and adolescents with Tourette’s disorder (TD). However, to date, a randomized controlled trial for aripiprazole oral solution has not been conducted despite being widely preferred by children. Therefore, we examined whether aripiprazole oral solution is effective for treating tics.

**Methods:**

All patients received a flexible dose of aripiprazole oral solution (1 mg/mL, range: 2–20 mg) with a starting dose of 2 mg. The target dose for patients weighing < 50 kg was 2, 5, and 10 mg/day, and that for patients weighing ≥ 50 kg was 5, 10, 15, and 20 mg/day. The primary efficacy endpoint was the mean change in the Yale Global Tic Severity Scale-total tic score (YGTSS-TTS) from baseline to week 8.

**Results:**

Of the 121 patients enrolled, 59 patients (96.7%) in the aripiprazole group and 53 patients (88.3%) in the placebo group completed the study. The aripiprazole group showed significantly greater improvement in the YGTSS-TTS from baseline to week 8 than the placebo group (least squares mean difference [95% confidence interval (CI)] −5.5 [95% CI − 8.4 to − 2.6]). At week 8, the response rate (i.e., percentage of patients with a Tourette’s Syndrome Clinical Global Impression-Improvement score of 1 or 2) of the aripiprazole group (86.4%) was significantly higher than that of the placebo group (56.6%; odds ratio: 3.6, *p* < 0.001). The incidence of treatment-emergent adverse events (TEAEs) reported in at least one patient was 86.9% in the aripiprazole group and 65.5% in the placebo group. All TEAEs were mild or moderate in severity. No serious adverse events or deaths occurred during the study.

**Conclusions:**

Our findings suggest that aripiprazole oral solution is an effective, well-tolerated, and safe treatment for children and adolescents with TD.

**Trial registration:**

ClinicalTrials.gov Identifier: NCT03487783. Registered 4 April 2018.

**Supplementary Information:**

The online version contains supplementary material available at 10.1186/s13034-024-00764-6.

## Introduction

Tourette’s Disorder (TD) is a neuropsychiatric condition that is characterized by the appearance of simple or complex tics, which are sudden, rapid, recurrent, and nonrhythmic stereotyped motor movements or vocalizations. Tics associated with TD become prominent in early childhood and worsen progressively, with severity peaking at 10 years of age [1]. Tics negatively impact the quality of life of children [2]. Thus, behavioral or pharmaceutical treatment may be considered for tics that cause discomfort or impair function [3–6].

Although the precise etiology and pathogenesis of TD are uncertain, atypical antipsychotics that selectively block dopamine D2 receptors have been shown to be effective for the treatment of tics [7, 8] and are now the primary treatment for tics alongside behavioral interventions. However, many antipsychotics are used off-label because there has been little development of new medications over the past 30 years. Furthermore, these medications can induce side effects, such as excessive sedation, which can impact academic development in children.

In China, several antipsychotics, including traditional Chinese medicines, are used for the treatment of tics, although the mechanisms underlying their effects are currently unknown [9] The incidence of problems, such as sedation and weight gain, is increasing with the use of these antipsychotics. Oral solution formulations are preferred over tablets in young children who have difficulty taking tablets.

Aripiprazole is an atypical antipsychotic with a unique mechanism of action: dopamine D2 receptor partial agonism [10]. This partial blocking action of dopamine is a key factor for childhood treatment. It improves tics, has a relatively favorable adverse event (AE) profile, and causes no prolactin elevation and limited sedation in pediatric and adolescent patients [11–13]. Randomized, double-blind, placebo-controlled studies of aripiprazole in TD patients conducted in South Korea and the United States have led to its approval for treating TD patients aged 6–18 years [14, 15]. Aripiprazole is currently the most frequently recommended antipsychotic for tic treatment [16–19] and has also been approved in the United States for the treatment of schizophrenia in adolescents (aged 13–17 years), bipolar mania in children and adolescents (aged 10–17 years), and irritability associated with autistic disorder in children and adolescents (aged 6–17 years) [20].

Aripiprazole oral tablets have shown efficacy in the suppression of tics and are generally well tolerated by TD patients [14, 15, 21–23]. However, it remains a Level B recommended drug for treating TD based on the results of few large randomized controlled trials (RCTs) [13, 24, 25].

To examine the efficacy of aripiprazole in treating TD, we conducted a large RCT of aripiprazole oral solution in Chinese children and adolescents with TD. Because difficulty taking tablets is common in children (a European survey reported that liquid is widely preferred by children aged < 12 years) [26], we used an oral solution of aripiprazole to consider treatment compliance in children. To the best of our knowledge, this is the first RCT to investigate the acute treatment of TD in children and adolescents using an aripiprazole oral solution.

## Methods

### Study design

In this phase 3, randomized, double-blind, placebo-controlled trial (ClinicalTrials.gov Identifier: NCT03487783), we recruited patients from 12 sites across China from May 2018 to December 2019. The study protocol and amendments, informed consent form, and patient recruitment materials were reviewed and approved by the ethics committee of each trial site. This study was conducted in compliance with the International Conference on Harmonisation Good Clinical Practice and applicable local laws and requirements.

The patients were randomized 1:1 to either the aripiprazole oral solution group or the matched placebo group to receive 8 weeks of treatment. The study comprised two phases: (1) a pretreatment phase of a maximum of 42 days of screening and washout (where applicable) and (2) a treatment phase with a baseline visit on day 0. Each patient received a flexible dose of aripiprazole oral solution (1 mg/mL), ranging from 2 to 20 mg/day. The starting dose for all randomized patients was 2 mg. The target dose for patients weighing < 50 kg was 2, 5, and 10 mg/day, and that for patients weighing ≥ 50 kg was 5, 10, 15, and 20 mg/day. Dose escalation was determined at each visit and was based on the patient’s global tic-related state and overall tolerability. The dose was titrated to the next dose if the patient had a Tourette’s Syndrome Clinical Global Impression-Improvement (TS-CGI-I) score of ≥ 3 (minimally improved) and a favorable tolerability assessment. The dose was maintained if the patient had a TS-CGI-I score of 1 (very much improved) or 2 (much improved) and adequate tolerability. The dose could be decreased for tolerability at any time at our discretion; however, the dose was ideally kept stable for the final 2 weeks of treatment.

### Patients

Written informed consent was obtained from all patients and their legal guardians. Patients aged 6–17 years who met the Diagnostic and Statistical Manual of Mental Disorders, Fourth Edition, Text Revision diagnostic criteria for TD and had a Yale Global Tic Severity Scale (YGTSS)-total tic score (TTS) of ≥ 22 at baseline were included in the study. Patients weighing < 15 kg were excluded. Patients with secondary tic symptoms accompanied by late-onset tics (tics with other causes, such as brain injury), Huntington’s chorea, neuroacanthocytosis, mental retardation, autism, low intelligence, bipolar disorder, schizophrenia, or depressive disorder were also excluded. Additionally, patients with comorbidities requiring drug therapy, such as attention deficit/attention-deficit hyperactivity disorder, obsessive-compulsive disorder, or oppositional defiant disorder, were excluded.

### Outcomes

The primary efficacy endpoint was the mean change in the YGTSS-TTS from baseline to week 8. The YGTSS is a valuable instrument for assessing tic severity in children, adolescents, and adults, and consists of a combination of motor and vocal tic severity scores (each on a scale of 0–5) across five dimensions: number, frequency, intensity, complexity, and interference [27]. All raters were trained on the YGTSS by qualified investigators at the investigators meeting, and newly assigned investigators were also trained during the study.

The secondary efficacy endpoints were the mean change in the percentage of the YGTSS-TTS from baseline to week 8, the response and partial response rates based on the TS-CGI-I scale at week 8, and the mean change in the Tourette’s Syndrome Clinical Global Impression-Severity of Illness (TS-CGI-S) [28] score from baseline to week 8. The response rate was defined as the percentage of patients with a TS-CGI-I score of 1 or 2, and the partial response rate was defined as the percentage of patients with a TS-CGI-I score of 3.

To assess AEs, we asked patients open questions about their overall feelings since treatment commencement and recorded all spontaneously reported AEs at each visit. Extrapyramidal symptoms (EPS) were assessed using the Simpson-Angus Scale (SAS), the Abnormal Involuntary Movement Scale (AIMS), and the Barnes Akathisia Rating Scale (BARS) at each visit. Vital signs, clinical laboratory test results, electrocardiogram (ECG), and mean weight change were measured at both randomization and endpoint.

### Statistical analyses

On the basis of previous data [14], to achieve 90% power and detect a treatment difference (standard deviation [SD]) of 5.35 (8.6) between aripiprazole and placebo according to the primary endpoint, we estimated the sample size to be 112. Considering other uncontrollable factors in the clinical trial, we estimated that 120 participants would be needed for randomization (*n* = 60 per group).

We used an analysis of covariance (ANCOVA) model, which was based on the last observation carried forward (LOCF) and full analysis set (FAS), as the primary analysis method. The model included the baseline value as the covariance and the treatment group, trial site, and baseline weight (≥ 50 kg vs. < 50 kg) as the fixed effects. The ANCOVA model was applied to increase the analysis efficacy through the addition of the fixed effects, and the LOCF method was used because it is the most commonly used analysis method to address missing data. Potential bias was limited because of our low dropout rate (we anticipated a dropout rate of 6.7% (4/60 patients) and 7.4% of patients dropped out of the study). The analyses for continuous secondary efficacy endpoints were based on the ANCOVA model, which included the baseline value as covariance as well as treatment group, trial site, and baseline weight (≥ 50 kg vs. < 50 kg) as the fixed effects. The response and partial response rates were analyzed using the Cochran–Mantel–Haenszel test, stratified by trial site. As one kind of subjective endpoint, the questionnaire might be influenced at site level, so the test was stratified by the trial site.

Randomized patients who received at least one dose of study medication were analyzed for safety (i.e., the safety set). Descriptive statistics were used to analyze AEs. The SAS, AIMS, and BARS scores were analyzed using an ANCOVA.

In the *post hoc* analysis, the percentage of patients with > 50% improvement in the YGTSS-TTS to reflect the response ratio versus placebo and CGI improvement and the mean change in the motor and vocal tic scores were analyzed using the Cochran–Mantel–Haenszel test for the proportion and an ANCOVA model for the change from baseline value. All analyses were performed using SAS version 9.4 (SAS Institute Inc., Cary, NC, USA), and *p* < 0.05 was considered significant.

## Results

### Patient characteristics and disposition

**Fig. 1:**
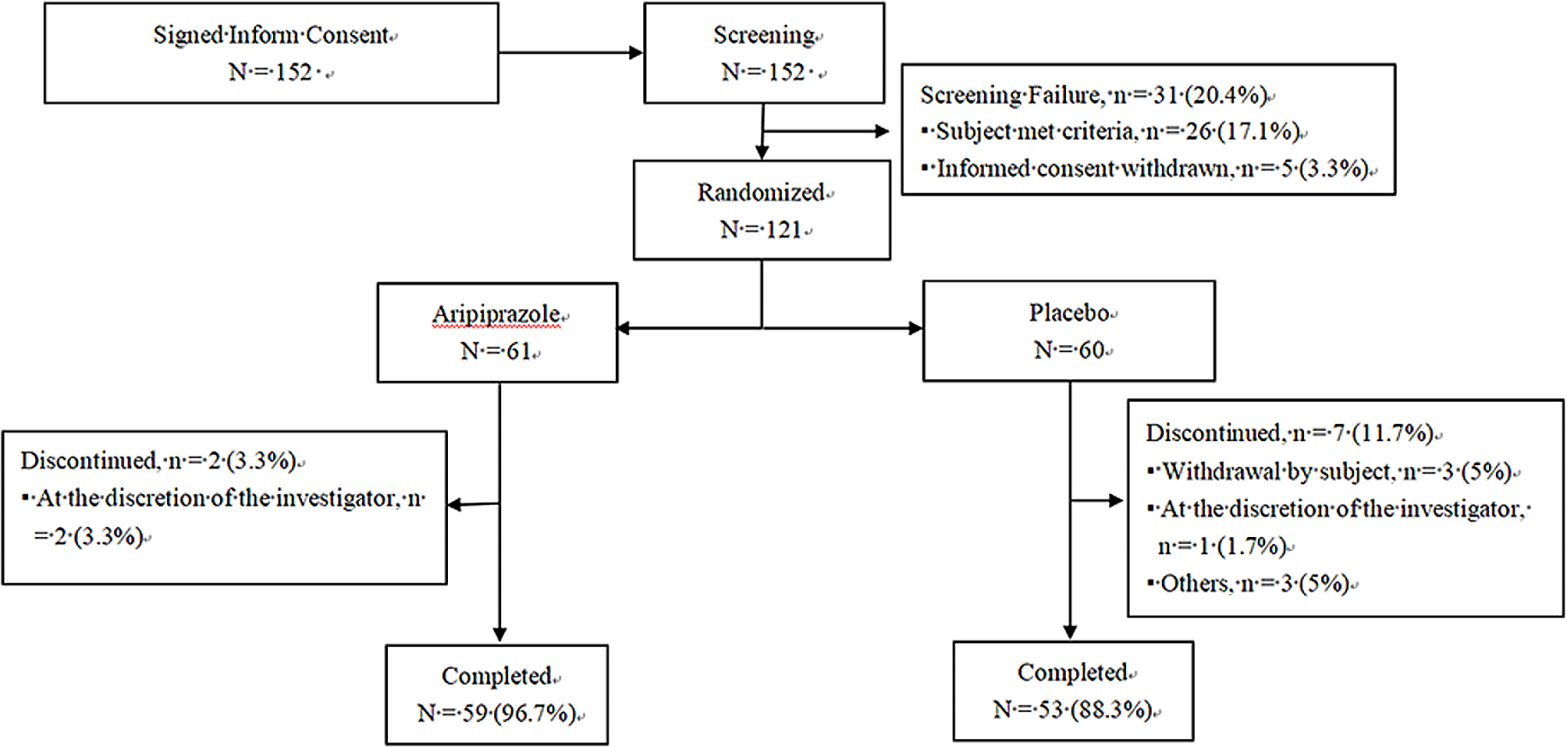
Patient disposition

Of the 121 patients enrolled in the study, 59 patients (96.7%) in the aripiprazole group and 53 patients (88.3%) in the placebo group completed the study (Fig. 1). The most common reason for discontinuation during the 8 weeks was our decision to withdraw the patient (3.3%) in the aripiprazole group and withdrawal by the patient (5%) in the placebo group. Demographics and baseline clinical characteristics were similar across the two groups (Table 1). Most patients were male and weighed < 50 kg. The mean age of the patients was 10.8 years. All patients were Chinese, primarily Han Chinese (96.7%).

**Table 1:** Demographics and baseline characteristics (intent to treat)

	Aripiprazole (*N* = 61)	Placebo (*N* = 60)
Age (year), mean (SD)	10.6 (2.5)	10.9 (2.7)
Male, n (%)	47 (77.0)	47 (78.3)
Ethnicity, n (%)		
Han	59 (96.7)	58 (96.7)
Others	2 (3.3)	2 (3.3)
Height (cm), mean (SD)	146.0 (15.6)	148.8 (15.4)
Weight (kg), mean (SD)	40.8 (14.3)	42.7 (16.1)
< 50 kg, n (%)	46 (75.4)	44 (73.3)
≥ 50 kg, n (%)	15 (24.6)	16 (26.7)
BMI (kg/m^2^), mean (SD)	18.5 (3.5)	18.6 (3.9)
Age of first diagnosis (year), mean (SD)	8.6 (2.8)	9.0 (2.7)
YGTSS-TTS, mean (SD)	31.6 (7.4)	32.6 (6.9)
YGTSS motor tic, mean (SD)	18.6 (3.5)	18.2 (4.0)
YGTSS vocal tic, mean (SD)	13.0 (5.3)	14.4 (4.9)
TS-CGI-S, mean (SD)	4.8 (0.9)	5.0 (0.9)

TS-CGI-S score ranges from 1 to 7; a higher score represents more severe symptoms

*BMI* body mass index, *SD* standard deviation, *YGTSS-TTS* yale global tic severity scale-total tic score, *TS-CGI-S* Tourette’s syndrome clinical global impression-severity of illness score

### Study medication

The mean exposure dosage for all aripiprazole patients, patients who weighed < 50 kg, and patients who weighed ≥ 50 kg was 6.8 (SD 2.8), 6.2 (SD 2.2), and 8.4 (SD 3.6) mg/day, respectively.

The distribution of aripiprazole dosage during the last week of treatment was as follows: for patients who weighed < 50 kg (*N* = 46), 10.9% had 2 mg/day (*n* = 5), 39.1% had 5 mg/day (*n* = 18), and 50% had 10 mg/day (*n* = 23); in patients who weighed ≥ 50 kg (*N* = 15), 6.7% had 2 mg/day (*n* = 1), 20% had 5 mg/day (*n* = 3), 40% had 10 mg/day (*n* = 6), 6.7% had 15 mg/day (*n* = 1), and 26.7% had 20 mg/day (*n* = 4; Supplementary Table 1).

The most frequent previous medications taken (≥ 5% in the aripiprazole or placebo group) were unspecified herbal and traditional Chinese medicine (19.7% of the aripiprazole group and 25.0% of the placebo group), benzamides (14.8% of the aripiprazole group and 15.0% of the placebo group), centrally acting sympathomimetics (8.2% of the aripiprazole group and 6.7% of the placebo group), and vitamin B-complex and other combinations (6.6% of the aripiprazole group and 8.3% of the placebo group). The most common antipsychotics (≥ 5% in the aripiprazole or placebo group) previously used to treat TD were tiapride/tiapride hydrochloride (8.2%/6.6% of the aripiprazole group and 6.7%/8.3% of the placebo group) and haloperidol (1.6% of the aripiprazole group and 6.7% of the placebo group). Previous use of anxiolytics to treat TD was 1.6 and 1.7% in the aripiprazole and placebo groups, respectively.

### Efficacy

**Fig. 2:**
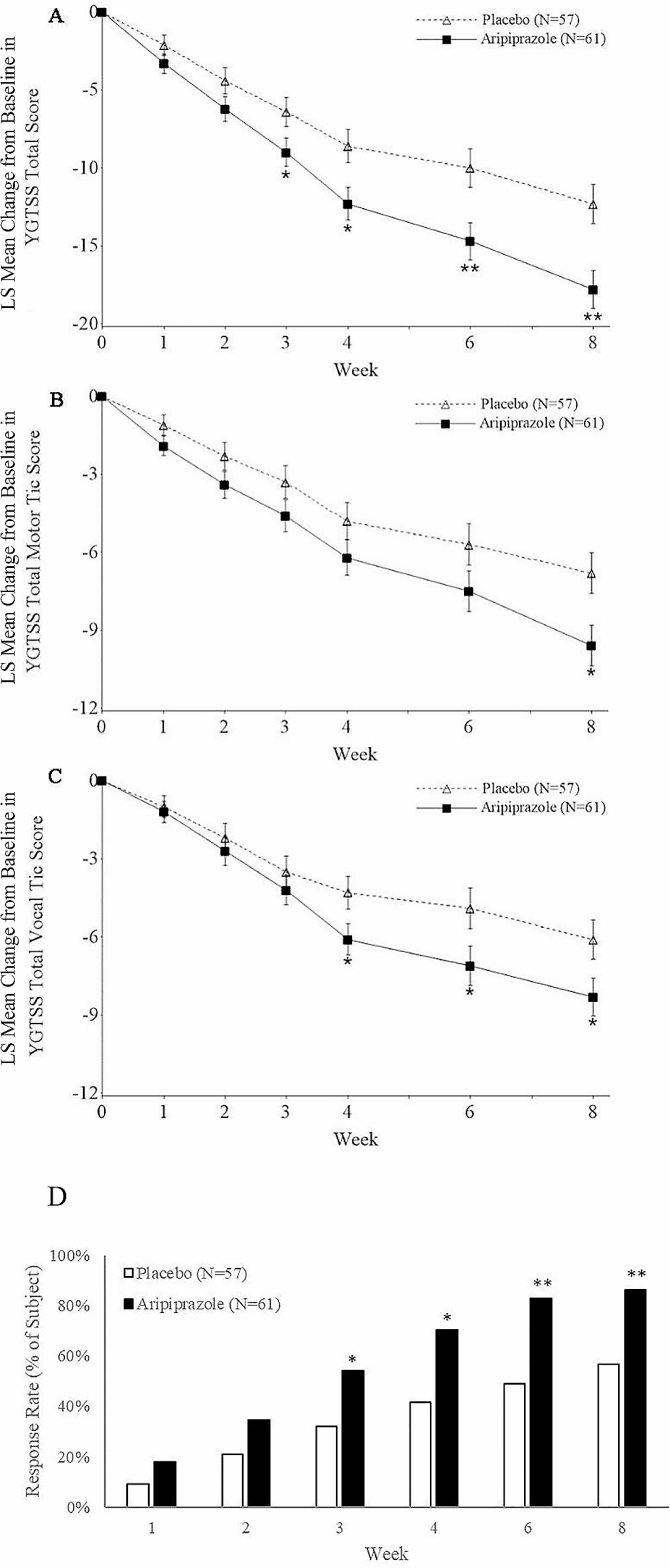
Yale Global Tic Severity Scale (YGTSS)-total tic score (TTS) (full analysis set [FAS], analysis of covariance [ANCOVA], and last observation carried forward [LOCF]) and response rate (FAS and observed cases [OCs]). ** A**–**C** Least squares mean (LSM) change from baseline for the YGTSS-TTS, YGTSS motor tic score, and YGTSS vocal tic score by week, calculated using ANCOVA. **A** LSM (standard error [SE]) change from baseline for the YGTSS-TTS. **B** LSM (SE) change from baseline for the YGTSS motor tic score. **C** LSM (SE) change from baseline for the YGTSS vocal tic score. Error bars represent the LSM ± SE. *p < 0.05, **p < 0.001. **D** Response rate, the percentage of patients with a Tourette’s Syndrome Clinical Global Impression-Improvement score of 1 or 2. *p < 0.05; **p < 0.001. *YGTSS* yale global tic severity scale, *TTS* total tic score, *FAS* full analysis set, *OC* observed cases, *ANCOVA* analysis of covariance, *LOCF* last observation carried forward, *LSM* least squares mean, *SE* standard error, *TS-CGI-I* Tourette’s syndrome clinical global impression-improvement

The aripiprazole group showed significantly greater improvement in the YGTSS-TTS than the placebo group at week 8 (least squares mean difference [95% confidence interval (CI)] −5.5 [95% CI − 8.4 to − 2.6]; Table 2). The significance difference was observed at week 3 (*p* < 0.05) and increased further at weeks 6 and 8 (Fig. 2A).

**Table 2:** Primary and secondary efficacy endpoints and yale global tic severity scale subscales (full analysis set)

	Aripiprazole (*N* = 61) *n* (%)	Placebo (*N* = 57) *n* (%)	*p* value
YGTSS-TS, LOCF			
Baseline, mean (SD)	31.6 (7.4)	32.7 (7.0)	
Change to week 8 (SE)	− 17.8 (1.2)	− 12.3 (1.3)	
Difference of LSM (95% CI)	− 5.5 (− 8.4, − 2.6)		< 0.001
YGTSS change rate, LOCF			
Change to week 8 (SE)	− 56.0% (3.7)	− 39.2% (3.8)	
Difference of LSM (95% CI)	− 16.7% (− 25.6, − 7.9)		< 0.001
Response rate, OC			
Week 8	86.4%	56.6%	
Odds ratio (95% CI)	3.6 (1.5, 8.9)		< 0.001
Partial response rate, OC			
Week 8	8.5%	34.0%	
Odds ratio (95% CI)	0.3 (0.1, 0.7)		< 0.001
*Post hoc* 50% response rate, OC			
Week 8	61.0%	37.7%	
Odds ratio (95% CI)	2.6 (1.2, 5.5)		0.015
TS-CGI-S, LOCF			
Baseline, mean (SD)	4.8 (0.9)	5.0 (0.9)	
Change to week 8 (SE)	− 2.1 (0.2)	− 1.4 (0.2)	
Difference of LSM (95% CI)	− 0.7 (− 1.1, − 0.3)		< 0.001
*Post ho*c YGTSS motor tic, LOCF			
Baseline, mean (SD)	18.6 (3.5)	18.4 (3.9)	
Change to week 8 (SE)	− 9.6 (0.8)	− 6.8 (0.8)	
Difference of LSM (95% CI)	− 2.8 (− 4.6, − 0.9)		0.004
*Post hoc* YGTSS vocal tic, LOCF			
Baseline, mean (SD)	13.0 (5.3)	14.4 (5.0)	
Change to week 8 (SE)	− 8.3 (0.7)	− 6.1 (0.8)	
Difference of LSM (95% CI)	− 2.3 (− 4.0, − 0.5)		0.012

Response ratio > 1 favors aripiprazole

YGTSS-TTS: score ranges from 0 to 50

TS-CGI-I and -S: scores range from 1 to 7

Response rate: percentage of the patients with a TS-CGI-I score of 1 (very much improved) or 2 (much improved)

Partial response rate: percentage of the patients with a TS-CGI-I score of 3 (minimally improved)

In the *post hoc* analyses, a 50% response rate was defined as a 50% decrease in YGTSS-TTS from baseline to endpoint

*LOCF* last observation carry forward, *SD* standard deviation, *SE* standard error, *LSM* least squares mean, *CI* confidence interval, *TS-CGI-I* Tourette’s syndrome clinical global impression-improvement score

Post hoc analyses of the mean change in motor and vocal tic scores showed significant differences (*p* < 0.05) for both types of tics between the aripiprazole and placebo groups at week 8 (Table 2; Fig. 2B and C). The vocal tic score differed significantly (*p* < 0.05) between the two groups from week 4 through to week 8.

The mean percent change of the YGTSS-TTS from baseline to week 8 was − 56.0% (standard error [SE] 3.7) and − 39.2% (SE 3.8) in the aripiprazole and placebo groups, respectively (*p* < 0.001; Table 2). The response rate of the aripiprazole group (86.4%) was significantly higher than that of the placebo group (56.6%) at week 8 (odds ratio 3.6, *p* < 0.001). However, the partial response rate was significantly lower in the aripiprazole group (8.5%) than in the placebo group (34.0%; odds ratio 0.3, *p* < 0.001; Table 2). The distribution of the response rate during the trial is shown in Fig. 2D, which shows the significant group difference beginning at week 3 and persisting through to week 8. The aripiprazole group also showed significantly greater improvement in the TS-CGI-S score than the placebo group from week 3 to week 8 (*p* < 0.001; Table 2).

In the post hoc analysis, we defined a 50% response rate as the percentage of patients who showed a > 50% decrease in the YGTSS-TTS from baseline to week 8. The aripiprazole group had a significantly greater 50% response rate than the placebo group from week 4 through to week 8 (*p* = 0.015, Table 2).

### Safety

#### AEs

The incidence of treatment-emergent AEs (TEAEs) reported in ≥ 1 patient was 86.9% in the aripiprazole group and 65.5% in the placebo group. Common TEAEs reported in the aripiprazole group were somnolence, lethargy, decrease in blood prolactin, influenza-like illness, tremor, and akathisia (Table 3). All TEAEs were mild or moderate in severity. No serious AEs or deaths occurred during the study.

**Table 3:** Treatment-emergent adverse events reported in ≥ 5% patients and extrapyramidal symptom scales (safety set)

	Aripiprazole (*N* = 61) *n* (%)	Placebo (*N* = 58) *n* (%)
Somnolence	11 (18.0)	8 (13.8)
Lethargy	10 (16.4)	5 (8.6)
Blood prolactin decreased	9 (14.8)	0
Influenza like illness	8 (13.1)	8 (13.8)
Tremor	8 (13.1)	2 (3.4)
Akathisia	7 (11.5)	1 (1.7)
Vomiting	5 (8.2)	9 (15.5)
Nausea	5 (8.2)	5 (8.6)
Stiff tongue	5 (8.2)	1 (1.7)
Vision blurred	5 (8.2)	0
Nasopharyngitis	4 (6.6)	4 (6.9)
Upper respiratory tract infection	4 (6.6)	4 (6.9)
Dizziness	4 (6.6)	3 (5.2)
Diarrhoea	4 (6.6)	2 (3.4)
Drooling	4 (6.6)	1 (1.7)
Dyskinesia	4 (6.6)	1 (1.7)
Blood insulin increased	4 (6.6)	1 (1.7)
Headache	3 (4.9)	4 (6.9)
Pyrexia	2 (3.3)	3 (5.2)

EPS scales	Parameters	Aripiprazole (*N* = 61)	Placebo (*N* = 58)	p value
SAS	LSM (SE)	0.2 (0.1)	0.0 (0.1)	
	Difference of LSM (95% CI)	0.2 (− 0.0, 0.4)		0.051
BARS	LSM (SE)	0.1 (0.1)	0.0 (0.1)	
	Difference of LSM (95% CI)	0.1 (− 0.1, 0.3)		0.288
AIMS	LSM (SE)	− 0.4 (0.1)	-0.3 (0.1)	
	Difference of LSM (95% CI)	− 0.1 (− 0.3, 0.1)		0.321

SAS total score ranges from 0 to 40

BARS total score ranges from 0 to 5

AIMS score ranges from 0 to 28

Higher scores represent a worse condition, and a reduction from baseline represents improvement

*EPS* extrapyramidal symptoms, *SAS* Simpson-angus scale, *BARS* Barnes akathisia rating scale, *AIMS* abnormal involuntary movement scale, *LSM* least squares mean, *SE* standard error, *CI* confidence interval

The incidence of the most common TEAEs (i.e., somnolence and lethargy) in the aripiprazole group by body weight (< 50 kg [*N* = 46] vs. ≥ 50 kg [*N* = 15]) was 21.7% (10/46) vs. 6.7% (1/15) for somnolence and 19.6% (9/46) vs. 6.7% (1/15) for lethargy. The incidence of the EPS-related TEAEs of tremor and akathisia were 10.9% (5/46) vs. 20.0% (3/15) and 8.7% (4/46) vs. 20.0% (3/15), respectively, and the incidence of decreased prolactin was 19.6% (9/46) vs. 0% (0/15).

No patients in the aripiprazole group withdrew from the study because of TEAEs. In the placebo group, the incidence of TEAEs leading to discontinuation was 3.4%.

The incidence of EPS-related TEAEs was 27.9% and 10.3% in the aripiprazole and placebo groups, respectively. There were no significant differences between the aripiprazole and placebo groups in the SAS, BARS, or AIMS scores (*p* = 0.051, *p* = 0.288, and *p* = 0.321, respectively; Table 3).

#### Laboratory data, body weight, vital signs, and ECG

Hematology, chemistry, urinalysis, vital signs, and ECG results showed no clinically meaningful differences between the two groups except for weight gain, prolactin level, and low-density lipoprotein (LDL) level.

Mean weight change from baseline to week 8 was 1.7 (SD 1.5) kg and 0.8 (SD 1.3) kg in the aripiprazole and placebo groups, respectively (*p* = 0.002). The ratios of patients with a weight increase of ≥ 7% (i.e., a potentially clinically relevant weight gain) from baseline to week 8 were 21.3 and 5.2% in the aripiprazole and placebo groups, respectively (*p* = 0.014). This ratio was not significantly different between groups until week 6.

The mean change in body mass index from baseline to week 8 was 0.5 (SD 0.7) kg/m^2^ and 0.2 (SD 0.6) kg/m^2^ in the aripiprazole and placebo groups, respectively (*p* = 0.003). The aripiprazole group had a larger mean prolactin reduction from baseline to week 8 (− 6.1 [SD 6.1] µg/L) than the placebo group (0.4 [SD 4.5] µg/L; *p* < 0.001). A slightly yet significantly greater increase in LDL levels was observed in the aripiprazole group (4.4 [SD 16.0] mg/dL) than in the placebo group (− 1.9 [SD 13.9] mg/dL; *p* = 0.034).

## Discussion

This study showed that flexible-dose aripiprazole oral solution was significantly more efficacious than placebo for treating tics, as measured using the YGTSS, from weeks 3 to 8. Our findings are consistent with the results of previous trials conducted in the United States and South Korea, which both reported reductions in tic frequency [14, 15]. The mean exposure dosages of aripiprazole in our study were 6.8 (SD 2.8), 6.2 (SD 2.2), and 8.4 (SD 3.6) mg/day for all patients, those who weighed < 50 kg, and those who weighed ≥ 50 kg, respectively, which indicates that central dosing for TD is approximately 5–10 mg/day.

A 25% reduction in the YGTSS-TSS is considered a clinically meaningful improvement and is consistent with a TS-CGI-I score of 1 or 2 [29]. Both the reduction in the YGTSS-TTS and the response rate were significantly greater and more clinically meaningful in the aripiprazole group than in the placebo group from week 3 through to week 8. Additionally, the 50% response rate, which is a more stringent response than the 25% response rate, was significantly higher in the aripiprazole group than in the placebo group from week 4 through to week 8. These results suggest that the 50% response rate, which reflects a robust treatment response, of our patients was similar to that of the a priori-defined response rate and demonstrates strictly the response ratio versus placebo; thus, the treatment improved patients’ quality of life. The robust improvement in the 50% response rate in both our study and previous RCTs may enable aripiprazole to be changed from a Level B to a Level A recommendation for treating TD, which would allow children and adolescents with TD to experience a better school life.

We also showed that aripiprazole is more effective than placebo in treating both motor and vocal tics at 8 weeks. This is consistent with the results of Salles et al. [15] and a study conducted in South Korea, although the latter study reported significant improvement in vocal tics only because of the small sample size [14]. Therefore, aripiprazole may be effective for both motor and vocal tics.

Numerous first- and second-generation antipsychotics are used to treat TD tics. One head-to-head study demonstrated that aripiprazole is as effective as risperidone for treating children and adolescents with tic disorder [30]. A recent systematic review reported that in patients with TD, 88.6% responded to aripiprazole, 68.9% responded to clonidine, and 62.5% responded to risperidone [6]. We showed a similar response rate to aripiprazole of 86.4% at week 8.

Several reviews have stated that there is moderately strong evidence to support the use of aripiprazole for the treatment of TD [13, 25]. Our RCT in a large sample of TD patients adds to such evidence. The European clinical guidelines for TD recommend aripiprazole because it has a more favorable AE profile than first- and second-generation antipsychotics [19]. Aripiprazole is currently the second most commonly recommended antipsychotic, after clonidine, for the treatment of tic disorders based on the latest clinical trial data [16]. Thus, aripiprazole may be considered one of the best treatments for TD.

Antipsychotics for the treatment of tic disorders are recommended because they cause few AEs, and any AEs that occur are mild [31]. Clonidine, an alpha-2 adrenergic agonist, is the most commonly prescribed medication for tic disorders [16] because it is better tolerated than other antipsychotic medications [31]. The increasing use of aripiprazole for treating tics [5] may be attributed to its partial agonist effect on the dopamine D2 receptor, which is one of the most important factors for brain development [32]. Moreover, aripiprazole has a favorable AE profile, which includes the lack of prolactin elevation in developing pediatric patients [14, 15, 24].

We used an aripiprazole oral solution, which was approved in 2005 and 2009 in the United States and Japan, respectively. The oral solution was used in this study to improve compliance in children. One patient who weighed < 40 kg was unable to take aripiprazole oral tablets during the follow-up period after study completion because the formulation was changed from an oral solution to tablets. An oral solution may thus be preferred over tablets in pediatric patients, as reported in a European survey [26].

In general, aripiprazole oral solution was tolerated as well as tablets. The AEs that occurred in our trial are largely consistent with those reported in other studies examining aripiprazole oral tablet as a treatment for schizophrenia, bipolar mania, and autistic disorder in children [33–36]. However, the incidence of TEAEs in the aripiprazole and placebo groups (86.9% and 65.5%, respectively) was higher than that reported in previous studies, although the severity was similar. Although none of the patients in the aripiprazole group discontinued the medication due to AEs, all four patients who required a dose decrease due to intolerability weighed less than 50 kg. In a study conducted in South Korea, there were no differences between aripiprazole and placebo groups except for weight gain-related AEs, although dose escalation was slower than that used in our study and was based on fortnightly assessments [14]. Given the higher rate of AEs observed in our study, we suggest slower dose titration for pediatric patients who have low body weight to reduce the risk of AEs.

Although the efficacy of aripiprazole was similar, there were small differences in AEs between our study and a previous trial conducted in the United States. Sedative-related AEs (e.g., sedation, somnolence, fatigue, and lethargy) were the main AEs reported in the US trial [15]; these occurred less frequently in our study. In contrast, we observed EPS-related AEs (e.g., tremor and akathisia) more frequently than in the US study. This discrepancy may be attributed to differences in the medical system between the United States and China.

Although comprehensive behavioral intervention is effective for reducing tics in children and adults [3, 4] aripiprazole appears to be slightly more effective [13]. In future tic treatment studies, aripiprazole should be directly compared with behavioral interventions or combination therapies.

In the treatment algorithm for TD, first-line treatment is typically non-pharmacological, followed by pharmacological interventions. However, many antipsychotics used to treat TD have side effects, which impact the quality of life of children, especially at school. Therefore, aripiprazole, a partial D2 receptor agonist, which has no impact on prolactin level and has minimal sedative effect may be the optimal pharmacological treatment for children and adolescents with TD. The present study has several limitations. First, the stringent inclusion criteria may limit the generalizability of our findings to all patients treated in clinical practice. Second, the study duration was short, and the age range of our patients was small (6–17 years). Further research on long-term aripiprazole use as an oral solution in TD patients with a wider age range and other chronic tic disorders is needed.

## Conclusion

Our findings demonstrate that aripiprazole oral solution is effective, well tolerated, and safe for the treatment of TD in children and adolescents. The results of three large RCTs, including our study, will allow aripiprazole to be changed from a Level B to a Level A recommended treatment for TD in children and adolescents. To enhance the generalizability of our data, further research on long-term aripiprazole use as an oral solution in TD patients with a wider age range and other chronic tic disorders is needed. In addition, aripiprazole should be directly compared with behavioral and combination therapy to ascertain the long-term efficacy and safety of aripiprazole.

### Supplementary Information

Supplementary File 1

## Data Availability

The datasets used and/or analysed during the current study are available from the corresponding author on reasonable request.
