# Supplementary material for: Randomized, double-blind, placebo-controlled trial of aripiprazole oral solution in children and adolescents with Tourette’s disorder
Source: Child Adolesc Psychiatry Ment Health. 2024 Jul 18;18:88. doi: 10.1186/s13034-024-00764-6 (PMC11264494; doi:10.1186/s13034-024-00764-6)
Supplement: Supplementary file 1 [file 13034_2024_764_MOESM1_ESM.docx]

**Supplementary Table 1** Prescribed dose during the last two weeks in aripiprazole group (safety set)

|  | < 50 kg  (N=46)  n (%) | ≥ 50 kg  (N=15)  n (%) |
| --- | --- | --- |
| 2 mg/day | 5 (10.9) | 1 (6.7) |
| 5 mg/day | 18 (39.1) | 3 (20.0) |
| 10 mg/day | 23 (50.0) | 6 (40.0) |
| 15 mg/day | 0 | 1 (6.7) |
| 20 mg/day | 0 | 4 (26.7) |
